# Diagnosis of patients with Lynch syndrome lacking the Amsterdam II or Bethesda criteria

**DOI:** 10.1186/s13053-023-00266-0

**Published:** 2023-10-20

**Authors:** Miguel Angel Trujillo-Rojas, María de la Luz Ayala-Madrigal, Melva Gutiérrez-Angulo, Anahí González-Mercado, José Miguel Moreno-Ortiz

**Affiliations:** 1 Doctorado en Genética Humana e Instituto de Genética Humana “Dr. Enrique Corona Rivera”, Departamento de Biología Molecular y Genómica, Centro Universitario de Ciencias de la Salud, Universidad de Guadalajara, Sierra Mojada #950, Col. Independencia, Guadalajara, C.P. 44340 Jalisco México; 2 Instituto de Genética Humana “Dr. Enrique Corona Rivera”, Departamento de Biología Molecular y Genómica, Centro Universitario de Ciencias de la Salud, Sierra Mojada #950, Col. Independencia, Guadalajara, C.P. 44340 Jalisco México; 3 Departamento de Ciencias de la Salud, Centro Universitario de los Altos, Universidad de Guadalajara, Av. Rafael Casillas Aceves #1200. Tepatitlán de Morelos, C.P. 47620 Jalisco, México

**Keywords:** Lynch Syndrome, Clinical criteria, Genetics aspects, Amsterdam, Bethesda

## Abstract

**Background:**

Lynch Syndrome (LS) is an autosomal dominant inheritance disorder characterized by genetic predisposition to develop cancer, caused by pathogenic variants in the genes of the mismatch repair system. Cases are detected by implementing the Amsterdam II and the revised Bethesda criteria, which are based on family history.

**Main body:**

Patients who meet the criteria undergo posterior tests, such as germline DNA sequencing, to confirm the diagnosis. However, these criteria have poor sensitivity, as more than one-quarter of patients with LS do not meet the criteria. It is very likely that the lack of sensitivity of the criteria is due to the incomplete penetrance of this syndrome. The penetrance and risk of developing a particular type of cancer are highly dependent on the affected gene and probably of the variant. Patients with variants in low-penetrance genes have a lower risk of developing a cancer associated with LS, leading to families with unaffected generations and showing fewer clear patterns. This study focuses on describing genetic aspects of LS cases that underlie the lack of sensitivity of the clinical criteria used for its diagnosis.

**Conclusion:**

Universal screening could be an option to address the problem of underdiagnosis.

**Supplementary Information:**

The online version contains supplementary material available at 10.1186/s13053-023-00266-0.

## Introduction

Lynch Syndrome (LS), previously known as hereditary non-polyposis colorectal cancer [1, 2], is an autosomal dominant inheritance disorder characterized by genetic predisposition to develop cancer in different organs, mainly in gastrointestinal and genitourinary systems [3, 4]. It is the most common cause of hereditary colorectal cancer (CRC) [5]. LS constitutes 2–4% of CRC cases [6, 7]. This predisposition is caused by the presence of germline pathogenic variants in at least one of the genes of the mismatch repair system (MMR): *MLH1*, *MSH2*, *MSH6* or *PMS2* [3, 4, 8]. The MMR system corrects errors that arise during DNA replication. The inactivation of these genes causes deficiencies in the mismatch repair system (dMMR) and a phenomenon known as microsatellite instability (MSI), which consists of an accumulation of alterations in the lengths of microsatellite regions [4, 9]. MSI is strongly associated with LS [10].

The most prevalent cancers in LS cases are colorectal and endometrial cancer (EC) [3]. One of the first steps to reach the diagnosis is the application of the Amsterdam II and revised Bethesda clinical criteria, which allow for selecting patients with a high risk of having LS, and who therefore must undergo further tests [11], such as immunohistochemical assays, MSI tests, and finally germline DNA analysis, to confirm the presence of pathogenic variants [12]. However, many of the affected individuals do not meet such criteria and are excluded from these analyses. These clinical criteria have low sensitivity; specifically, they seem to be less sensitive in the detection of cases associated with certain genes, such as pathogenic variants in *MSH6* [13]. This study aims to highlight the genetics and molecular characteristics of patients who do not meet such criteria, but who can be considered as LS.

## Clinical criteria

In 1991, the Amsterdam I criteria were published, which were established as the minimal clinical criteria for the identification of patients and families at high risk of having LS [14, 15]. These criteria were created to provide common guidelines for the selection of families for research and for the comparison of results between studies; due to this, the criteria prioritized specificity more than sensitivity. The application of these criteria to the clinical diagnosis can lead to the exclusion of more than 50% of cases [15, 16]. The creators clarified that they are not intended to serve as a guide to exclude suspicious families that might require genetic counseling and molecular analysis. Because the Amsterdam I criteria are very rigid and do not consider extracolonic tumors associated with LS, they were modified, and then named the Amsterdam II criteria (Table 1) [15]. Currently, LS has been associated with an increased risk of developing extracolonic cancers, such as EC, ovary [17], upper tract urothelial carcinoma [18], prostate [19], bladder [20], small intestine, stomach [21], hepatobiliary tract, and pancreas, among others [22, 23].

**Table 1:** Amsterdam I and II clinical criteria [14, 15]

**Amsterdam I (1991)**
There must be at least three relatives with colorectal cancer; all the following criteria must be present: 1-One of the cases must be a first-degree relative of the other two 2-At least two successive generations must be affected 3-At least one case must have been diagnosed before the age of 50 4-Familial Adenomatous Polyposis must be excluded 5-Tumors must be verified by pathological examination
**Amsterdam II (1999)**
There must be at least three relatives with an HNPCC-associated cancer (colorectal cancer, cancer of the endometrium, small intestine, ureter, or renal pelvis) 1-One of the cases must be a first-degree relative of the other two 2-At least two successive generations must be affected 3-At least one must be diagnosed before the age of 50 4-Familial Adenomatous Polyposis should be excluded in cases of colorectal cancer 5-Tumors must be verified by pathological examination

HNPCC: Hereditary nonpolyposis colorectal cancer

These criteria have been shown to have low sensitivity in identifying carriers of LS-causing variants. This motivated the creation of the Bethesda criteria, with broader aspects that allow the identification of a greater proportion of affected people [4, 24]. These were later revised and modified. The Bethesda criteria make it possible to identify individuals who should be evaluated for MSI, and thus help identify patients with LS (Table 2) [24, 25]. As previously mentioned, MSI is a hallmark of LS, since it is a phenomenon present in 95% of cases [26]; however, it is not exclusive, since approximately 80% of tumors with dMMR/MSI are sporadic [27].

**Table 2:** Bethesda criteria [24, 25, 28]

**Original Bethesda Criteria (1997)**
Tumors from individuals should be evaluated for MSI in the following situations: 1-Individuals with cancer in families that meet the Amsterdam criteria 2-Individuals with two HNPCC-related cancers, including synchronous and metachronous colorectal cancers or associated extracolonic cancers 3-Individuals with colorectal cancer and a first-degree relative with colorectal cancer and/or an extracolonic cancer associated with HNPCC and/or a colorectal adenoma; one of the cancers diagnosed before the age of 45 and the adenoma diagnosed before the age of 40 4-Individuals with colorectal cancer or endometrial cancer diagnosed before the age of 45 5-Individuals with right-sided colorectal cancer with an undifferentiated pattern (solid/cribriform) on histopathology diagnosed before the age of 45 years 6-Individuals with signet ring cell colorectal cancer diagnosed before the age of 45 7-Individuals with adenomas diagnosed before the age of 40
**Revised Bethesda Criteria (2004)**
Tumors from individuals should be evaluated for MSI in the following situations: 1- Colorectal cancer diagnosed in a patient under 50 years of age 2-Presence of synchronous or metachronous colorectal tumors or other tumors associated with HNPCC, regardless of age. 3- Colorectal cancer with MSI-H histology diagnosed in a patient under 60 years of age 4- Colorectal cancer or a tumor associated with HNPCC diagnosed in one or more first-degree relatives, with one of the cancers diagnosed before the age of 50 years 5- Colorectal cancer or HNPCC-associated tumors diagnosed in two or more first- or second-degree relatives, regardless of age

MSI: Microsatellite instability. HNPCC: Hereditary nonpolyposis colorectal cancer. MSI−H: Microsatellite instability high

## Sensitivity and specificity of clinical criteria

The Amsterdam II criteria have a high specificity of up to 98% (27); however, they have low sensitivity, between 27% and 42% [16, 29]. In a particular study, out of 312 patients with LS, only 41 (14%) met the Amsterdam I criteria, 85 (27%) met the Amsterdam II, and 214 (69%) met the revised Bethesda criteria (at least one criterion) [16] (Table 3). The revised Bethesda criteria generally have higher sensitivity (82–95%) compared to the Amsterdam criteria but have lower specificity (77–93%) [27]. This scenario demonstrates that there are patients who have mutations in the MMR genes and who do not meet such clinical criteria [16]. There are also patients who meet the Amsterdam criteria and do not have identifiable mutations in the MMR genes [29]. In the latter case, a Lynch-like syndrome would be suspected, in which patients may meet the Amsterdam and Bethesda criteria, and present MSI and the absence of MMR proteins; however, these patients do not have identified germline mutations in the genes that encode proteins of the MMR system, and proposed explanations of this phenotype include the presence of cryptic or rare germline mutations in MMR genes, or even pathogenic germline mutations in genes like *MUTYH*, *POLE* and *POLD1*, the absence of the gene products of which could affect the MMR system [30].

**Table 3:** Sensitivity of clinical criteria in different studies

Country/authors	Patients with LS	Amsterdam I	Amsterdam II	Revised Bethesda criteria^1^	Cancer type
United States of America (USA), Finland [16]	312	41 (13%)	85 (27%)	214 (69%)	Colorectal cancer
Norway [13]	514	49 (10%)	79 (15%)	*	Colorectal cancer
China [31]	6	*	2 (33%)	2 (33%)	Endometrial cancer
Canada [32]	13	*	2 (15%)	9 (69%)	Endometrial cancer
Australia [33]	3	0 (0%)	0 (0%)	0 (0%)	Endometrial cancer
USA [34]	8	*	1 (13%)	0 (0%)	Endometrial cancer
USA [34]	16	*	8 (50%)	12 (75%)	Colorectal cancer
Korea [35]	30	*	20 (67%)	*	Endometrial cancer
Spain [36]	14	*	7 (50%)	12 (86%)	Colorectal cancer
USA [37]	5	*	1 (20%)	2 (40%)	Colorectal cancer
Scotland [29]	38	*	16 (42%)	36 (95%)	Colorectal cancer
Portugal [38]	4	*	0 (0%)	3 (75%)	Colorectal cancer
China [39]	93	*	9 (10%)	76 (82%)	Colorectal cancer

LS: Lynch Syndrome. 1At least one criterion. *Undetermined

## MMR genes

The proteins encoded by MMR genes have the function of correcting single-base pairing errors and loops caused by insertions or deletions that may occur during replication. When this correction system does not function properly, a mutator phenotype is produced that has its origin in a phenomenon known as MSI [40]. Microsatellites are short tandemly repeated sequences of 1–6 base pairs that are distributed throughout the genome (in coding and non-coding regions) and represent 3% of the human genome [41]. Microsatellites accumulate errors when the DNA polymerase does not bind efficiently to these repetitive sequences [42]. MSI consists of an alteration in the length of these repetitive regions due to a dMMR caused by inactivating mutations in MMR genes [43]. When the MMR is functional, these errors are recognized and corrected. In the presence of pathogenic variants in MMR genes, this system does not adequately carry out this repair function, allowing cells to maintain and replicate their mutations and acquire additional mutations (mutator phenotype) [42]. This mutator phenotype causes frameshift mutations in genes related to cancer development [44], since these genes contain microsatellite regions in their coding sequence, and this makes them susceptible to mutations due to dMMR [43].

Germline pathogenic variants in MMR genes underlie this syndrome; specifically, variants in the *MLH1* and *MSH2* genes represent 90% of all LS-causing variants, with *MSH6* contributing 7–10% and *PMS2* less than 5% [1]. Different alterations, such as the absence of *MSH2* expression due to a deletion of the *EPCAM* gene [45], and epimutation in the promoter region of *MLH1*, can be found [46]. In 2016, a review was conducted of the International Society for Gastrointestinal Hereditary Tumors (InSiGHT) database, which database of variants associated with LS has been maintained since 1996. According to this author, the variants in *MLH1*, *MSH2*, *MSH6* and *PMS2* represent 40%, 34%, 18%, and 8%, respectively, of the 3,000 germline variants of MMR genes deposited in this database [40]. Most variants in MMR genes appear to be inherited, and a very low proportion are *de novo* mutations [47]. Since the penetrance of pathogenic variants in MMR genes is less than 100%, some individuals with a predisposing variant in any of the MMR genes may never develop CRC [1].

## Penetrance and risk of cancer

CRC penetrance varies in patients who carry variants in MMR genes [48, 49]. Penetrance can be influenced by several variables, such as the specific variant, the affected gene, the sex of the carrier, lifestyle aspects, and other genetic characteristics [49]. Other studies use different measures of CRC risk, including cumulative penetrance, relative risks, or standardized incidence rates. Additionally, for heterozygous carriers with LS, the risk of CRC has been estimated considering age, sex, and affected genes [48]. This variability calls into question the average cumulative risk that is usually used in clinical practice, and it is very possible that it does not apply to all families. In a different study, analyzing families from Australia, New Zealand, North America and Europe, the variation in penetrance for CRC was estimated between carriers of pathogenic variants in the same gene by sex and geographic location; the variation was greater for carriers of variants in *MLH1* and *MSH2*, with 7–56% of carriers having a penetrance for CRC of less than 20%, 9–44% having a penetrance greater than 80%, and only 10–19% having a penetrance of 40–60%. The carriers of variants in *MLH1* and *MSH2* presented a higher penetrance on average, and the carriers of variants in *PMS2* presented a lower one [49]. Regarding the relationship between specific genes and tumor types, it has been observed that families with variants in *MSH2* have more extracolonic cancers than carriers of variants in *MLH1*. In turn, families carrying variants in *MSH6* develop cancer at a later age and have a higher risk of developing EC [4].

According to a prospective study that included 6,350 participants, pathogenic variants in *MLH1* and *MSH2* have high penetrance. In these patients, the lifetime risk of developing CRC is approximately 50%, with similar risks for endometrial and ovarian cancer. In contrast, carriers of variants in *MSH2* with advanced age had a higher risk of developing brain cancer, upper urinary tract and upper gastrointestinal tract, and more frequently prostate cancer [50]. Variants in *MSH6* cause a high risk of EC but a low risk of CRC in both genders. On the other hand, variants in *PMS2* did not confer a significant risk of cancer. The marked difference in cancer risks associated with MMR genes seems to justify the proposal to divide LS cases into four different hereditary cancer syndromes, each with its own clinical characteristics and potentially with specific management approaches [50]. These risk differences have serious implications for patient management since the current clinical practice guidelines in many countries are based on them [49].

## Sub-diagnosis of Lynch Syndrome

CRC and EC at an early age can often be a clue to diagnosis, since they are the most common tumors associated with LS. The first step in making the diagnosis is the application of the Amsterdam II clinical criteria and the revised Bethesda criteria, which are used to select individuals for further molecular testing [11]. However, there are studies that suggest that screening using these criteria could miss more than one-quarter of LS cases [51], and that is why most LS cases are undiagnosed. This fact takes on relevance when considering the frequency of this syndrome. In the United States, approximately 829,747 cases of LS are estimated, and of these, only 1.2% are diagnosed [52]. Another study conducted on 5,744 families from the USA, Canada and Australia estimated that 1 in 279 people carry a variant in one of the genes of the MMR system [53]. On the other hand, the revised Bethesda criteria have a poor performance in the identification of carriers of mutations in *MSH6* and, to a lesser extent, mutations in *PMS2* and *MSH2* genes [16]. In one study, it was found that the Amsterdam I and II and revised Bethesda criteria are not sensitive enough to detect patients with LS with variants in *MSH6* and *PMS2*. This study found that only 38% of families with *MSH2* variants, 12% of families with *MSH6* variants, 78% of families with *MLH1* variants, and 25% of families with *PMS2* variants met the criteria of Amsterdam I, while only 62%, 48%, 87% and 38%, respectively, met those of Amsterdam II. The Amsterdam criteria and each of the Bethesda criteria were inadequate in identifying families carrying mutations in *MSH6*. This suggests that *MSH6* mutations may be more common than currently assumed (See Supplementary Tables 1, Additional File 1) [13].

Comparing the accuracy of various screening techniques for LS in patients with EC, 6 patients (6/111) with LS were found, of which only 2 (33.33%) met the Amsterdam II criteria and the revised Bethesda criteria. Of those that did not meet the clinical criteria, three presented variants in *MSH6* and one in *MSH2* [31]. In another study, of 147 patients with EC associated with LS, only 84 (57.1%) fulfilled the family history screening criteria [54].

## Other guidelines and universal screening

In addition to the Amsterdam II and revised Bethesda criteria, other guidelines and recommendations have emerged for the screening of patients with LS. The Jerusalem guidelines recommend screening all CRC patients < 70 years of age by immunohistochemistry (for the MMR proteins) or an MSI test, regardless of whether they meet clinical criteria [55]. The Mallorca guidelines from the European Hereditary Tumour Group (EHTG) and European Society of Coloproctology (ESCP) recommend testing all CRC patients with immunohistochemistry or MSI testing, regardless of age [56].

Due to the high prevalence of carriers of variants in the MMR genes (estimated at 1 in 300) [56], and due to the lack of sensitivity of clinical criteria, universal screening for LS has been proposed, which refers to performing MSI and immunohistochemical tests on all patients with CRC [38, 57–59]. Regarding the universal screening of LS cases among cases of EC (the second most frequent), this has been inconsistent in many countries, and no consensus has been reached; however, some authors recommend including EC in universal screening for LS [34, 60]. It has been reported that routine LS screening in EC patients aged ≤ 70 years is a cost-effective strategy [61].

Currently, the Amsterdam II and revised Bethesda criteria are still widely used around the world. According to a study conducted in 12 countries in the Middle East and North Africa, the selection of families for genetic testing is based on these clinical criteria in most of the countries surveyed. Clinical criteria were used for the identification of LS in 8 of the 12 countries, and only 1 country offered systematic screening of tumors. In addition, the institutions that offer genetic diagnostic services in these countries are limited. Furthermore, it is suspected that in these countries, most families with LS are unidentified [62]. In Latin America, family history has been the main strategy for identifying patients at risk of LS, specifically in countries such as Brazil, Mexico, Paraguay, and Peru [63]. Groups such as the United States Multi-Society Task Force on Colorectal Cancer and the American Gastroenterological Association recommend the use of universal testing in all cases of CRC with immunohistochemistry and/or MSI tests [64].

## Conclusion

It is important that people with LS are diagnosed, since by determining their status, they can be offered proper management and can take prophylactic measures. All the evidence indicates that the clinical criteria should be reviewed, since they leave out many patients with LS. This lack of sensitivity is due to many reasons; clinical criteria are based on family history, however, in clinical practice, the collection of family history is often poor. Additionally, people with variants in low-penetrance genes, such as *MSH6* and *PMS2*, have a lower risk of developing a cancer associated with LS, leading to families with unaffected generations and showing fewer clear patterns. Most of the variants included in this review are reported in *MSH6*, *MSH2* and *PMS2*, genes that have been associated with lower penetrance. The risk of cancer varies greatly depending on the affected gene, gender, and age, so this information should be part of the management guidelines for patients with LS. Finally, universal screening has been suggested by performing MSI and immunohistochemistry in all patients with CRC, since these tests have been shown to have greater sensitivity for the detection of patients with LS compared with clinical criteria.

### Electronic supplementary material

Below is the link to the electronic supplementary material.

Supplementary Material 1

## Data Availability

All data and material are available in the cited literature.

## List of abbreviations

CRC: Colorectal cancer
dMMR: Deficiency in the mismatch repair system
EC: Endometrial cancer
EHTG: European Hereditary Tumour Group
ESCP: European Society of Coloproctology
HNPCC: Hereditary nonpolyposis colorectal cancer
InSiGHT: International Society for Gastrointestinal Hereditary Tumors
LS: Lynch Syndrome
MMR: Mismatch Repair
MSI: Microsatellite instability
MSI-H: Microsatellite instability high
USA: United States of America
