# Supplementary material for: Diagnosis of patients with Lynch syndrome lacking the Amsterdam II or Bethesda criteria
Source: Hered Cancer Clin Pract. 2023 Oct 20;21:21. doi: 10.1186/s13053-023-00266-0 (PMC10589993; doi:10.1186/s13053-023-00266-0)
Supplement: Supplementary file 1 — Supplementary Material 1 [file 13053_2023_266_MOESM1_ESM.docx]

**Table 1. Variants in patients with Lynch Syndrome who do not meet Amsterdam II and/or revised Bethesda criteria.**

| Country/authors | Patients with LS | Cancer type | Molecular findings |
| --- | --- | --- | --- |
| Norway [1] | 514 | Colorectal cancer | ***MSH2*** |
|  |  |  | c.229_230delAG; p.Ser77CysfsX3 |
|  |  |  | c.815C→T |
|  |  |  | c.942+3A→T |
|  |  |  | c.1076+1G→A |
|  |  |  | c.1277-2A→G |
|  |  |  | c.1759G→C |
|  |  |  | c.1979A→G |
|  |  |  | c.142G→T; p.Glu48X |
|  |  |  | c.226C→T; p.Gln76X |
|  |  |  | c.1857T→G; p.Tyr619X |
|  |  |  | c.2038C→T; p.Arg680X |
|  |  |  | c.2275G→T; p.Gly759X |
|  |  |  | c.1-?_366+?del |
|  |  |  | c.1-?_1076+?del |
|  |  |  | c.1-?_1661+?del |
|  |  |  | c.571_573delCTC; p.Leu191del |
|  |  |  | ***MSH6*** |
|  |  |  | c.1943delG; p.Ser648MetfsX5 |
|  |  |  | c.3261dupC; p.Phe1088LeufsX5 |
|  |  |  | 3261delC; p.Phe1088ProfsX2 |
|  |  |  | c.3804dupA; p.Cys1269MetfsX5 |
|  |  |  | c.3647-2A→C |
|  |  |  | c.467C→G; p.Ser156X |
|  |  |  | c.718C→T; p.Arg240X |
|  |  |  | c.1444C→T; p.Arg482X |
|  |  |  | c.2731C→T; p.Arg911X |
|  |  |  | c.3991C→T; p.Arg1331X |
|  |  |  | c.2302_2304delCCT; p.Pro768del 4/14 |
|  |  |  | ***PMS2*** |
|  |  |  | c.736_741delCCCCCTinsTGTGTGTGAAG; p.Pro246CysfsX2 |
|  |  |  | c.2382dupT; p.Gly795TrpfsX29 |
|  |  |  | c.537+1G→T |
|  |  |  | c.989-1G→T |
|  |  |  | ***MLH1*** |
|  |  |  | c.790+1G→A |
|  |  |  | c.1731G→C |
|  |  |  | c.1732-?_1896+?del |
| China [2] | 6 | Endometrial cancer | ***MSH2*** |
|  |  |  | c.1813delG; p.Val605Leufs |
|  |  |  | ***MSH6*** |
|  |  |  | c.C742T; p.Arg248Ter |
|  |  |  | c.C3103T; p.Arg1035Ter |
|  |  |  | c.2598_2602delAGTAA; p.Lys866Asnfs |
| Canada [3] | 12 | Endometrial cancer | ***MSH2*** |
|  |  |  | c.882dupT |
|  |  |  | c.1786__1788delAAT; p.Asn596del |
|  |  |  | c.998G>A; p.Cys333Tyr |
|  |  |  | ***MSH6*** |
|  |  |  | c.4001G>A; p.Arg1334Gln |
|  |  |  | c.2300C>T; p.Thr767Ile |
|  |  |  | c.1139_1143delATGAG; p.Asp380fs |
|  |  |  | ***PMS2*** |
|  |  |  | Exon 6-8 deletion |
|  |  |  | c.137G>T; p. Ser46Ile |
|  |  |  | ***MLH1*** |
|  |  |  | Exon 6 deletion |
|  |  |  | c.454-13A>G |
| Australia [4] | 3 | Endometrial cancer | ***MSH2*** |
|  |  |  | chr2:g.47656969C>T |
|  |  |  | ***PMS2*** |
|  |  |  | chr7:g.6017220G>A |
|  |  |  | chr7:g.6026664G>A |
| USA [5] | 8 | Endometrial cancer | ***MSH2*** |
|  |  |  | c.1251_ 1268del18insAGTT |
|  |  |  | c.1950dupT |
|  |  |  | ***MSH6*** |
|  |  |  | c.3802-7_ –4delTCTT |
|  |  |  | 5’UTR_EX1del |
|  |  |  | c.1367G>A |
|  |  |  | nc.2731C>T |
|  |  |  | ***MLH1*** |
|  |  |  | c.2224C>T |
|  |  |  | c.1975C>T |
| USA [5] | 16 | Colorectal cancer | ***MSH6*** |
|  |  |  | c.3939_3957dup19 |
|  |  |  | c.10C>T |
|  |  |  | c.3202C>T |
|  |  |  | ***PMS2*** |
|  |  |  | c.1021delA |
|  |  |  | c.137G>T |
|  |  |  | 1407ins46 |
|  |  |  | ***MLH1*** |
|  |  |  | Germline *MLH1* promoter methylation |
| Korea [6] | 30 | Endometrial cancer | ***MSH2*** |
|  |  |  | c.1276 + 1G>A |
|  |  |  | c.187delG |
|  |  |  | c.792 + 1del |
|  |  |  | c.942 + 3A>T |
|  |  |  | ***MSH6*** |
|  |  |  | c.3261dupC |
|  |  |  | c.3226C>T |
|  |  |  | c.3489dup |
|  |  |  | ***PMS2*** |
|  |  |  | c.1738A>T |
|  |  |  | ***MLH1*** |
|  |  |  | c.1878_1881del |
|  |  |  | c.1036C>T |
| Spain [7] | 14 | Colorectal cancer | ***MSH2*** |
|  |  |  | c.2635-3C>T and c. 2635-5T>C |
|  |  |  | c.687dupA; p.Ala230fsX2 |
|  |  |  | c.1165C>T; p.Arg389X |
|  |  |  | c.2131C>T; p.Arg711X |
|  |  |  | c.1077-?_1276+? del; Arg359_Gly426> ArgfsX17 |
|  |  |  | ***MSH6*** |
|  |  |  | c.3477C>A; p.Tyr1159X |
|  |  |  | ***MLH1*** |
|  |  |  | c.1668-1G>A; p.Ser556ArgfsX14 |
| Scotland [8] | 38 | Colorectal cancer | ***MSH2*** |
|  |  |  | EX1_6del |
|  |  |  | c.1A>C; p.Met1Leu |
|  |  |  | c.754C>T; p.Q252X |
|  |  |  | c.942+3A>T |
|  |  |  | c.1320_1321insA; p.L440fsX442 |
|  |  |  | c.1786_1788delAAT; p.N596del |
|  |  |  | c.2634+5G>C |
|  |  |  | ***MSH6*** |
|  |  |  | c.3261_3262insC; p.F1088fsX1092 |
|  |  |  | c.3516_3527del12; p.V1173_R1176del |
|  |  |  | c.3519_3520insA; p.V1173fsX1175 |
|  |  |  | c.3840_3846delGGAGACT; p.Q1280fsX1324 |
|  |  |  | c.3958_3959ins19; p.A1320fsX1324 |
|  |  |  | ***MLH1*** |
|  |  |  | c.3G>A; p.Met1Ile |
|  |  |  | c.116G>T |
|  |  |  | c.116 +1G>A |
|  |  |  | c.588+1G>T |
|  |  |  | c.1017_1018delC; p.S339fsX366 |
|  |  |  | c.2041G>A; p.A681T |
|  |  |  | c.2099-2102delAGCA; p.Q700fs[X722] |
| Portugal [9] | 4 | Colorectal cancer | ***MSH2*** |
|  |  |  | c.388_389del; p.Gln130ValfsTer2 |
|  |  |  | ***PMS2*** |
|  |  |  | Deletion exons 1 to 14 (c.(?-87)_(2445+1_2446-1)del) |
|  |  |  | Deletion exons 12 to 14 (c.(2006+1_2007-1)_(2445+1_2446- 1)del) |
|  |  |  | ***MLH1*** |
|  |  |  | c.2041G>A; p.(Ala681Thr) |

^LS: Lynch Syndrome.^

1. Sjursen W, Haukanes BI, Grindedal EM, Aarset H, Stormorken A, Engebretsen LF, Jonsrud C, Bjørnevoll I, Andresen PA, Ariansen S, Lavik LA, Gilde B, Bowitz-Lothe IM, Maehle L, Møller P. Current clinical criteria for Lynch syndrome are not sensitive enough to identify MSH6 mutation carriers. J Med Genet. 2010;doi: 10.1136/jmg.2010.077677.
2. Chao X, Li L, Wu M, Ma S, Tan X, Zhong S, Bi Y, Lang J. Comparison of screening strategies for Lynch syndrome in patients with newly diagnosed endometrial cancer: a prospective cohort study in China. Cancer Commun (Lond). 2019;doi: 10.1186/s40880-019-0388-2.
3. Lawrence J, Richer L, Arseneau J, Zeng X, Chong G, Weber E, Foulkes W, Palma L. Mismatch Repair Universal Screening of Endometrial Cancers (MUSE) in a Canadian Cohort. Curr Oncol. 2021;doi: 10.3390/curroncol28010052.
4. Najdawi F, Crook A, Maidens J, McEvoy C, Fellowes A, Pickett J, Ho M, Nevell D, McIlroy K, Sheen A, Sioson L, Ahadi M, Turchini J, Clarkson A, Hogg R, Valmadre S, Gard G, Dooley SJ, Scott RJ, Fox SB, Field M, Gill AJ. Lessons learnt from implementation of a Lynch syndrome screening program for patients with gynaecological malignancy. Pathology. 2017;doi: 10.1016/j.pathol.2017.05.004.
5. Adar T, Rodgers LH, Shannon KM, Yoshida M, Ma T, Mattia A, Lauwers GY, Iafrate AJ, Hartford NM, Oliva E, Chung DC. Universal screening of both endometrial and colon cancers increases the detection of Lynch syndrome. Cancer. 2018;doi: 10.1002/cncr.31534.
6. Kim YN, Kim MK, Lee YJ, Lee Y, Sohn JY, Lee JY, Choi MC, Kim M, Jung SG, Joo WD, Lee C. Identification of Lynch Syndrome in Patients with Endometrial Cancer Based on a Germline Next Generation Sequencing Multigene Panel Test. Cancers (Basel). 2022;doi: 10.3390/cancers14143406.
7. Pérez-Carbonell L, Ruiz-Ponte C, Guarinos C, Alenda C, Payá A, Brea A, Egoavil CM, Castillejo A, Barberá VM, Bessa X, Xicola RM, Rodríguez-Soler M, Sánchez-Fortún C, Acame N, Castellví-Bel S, Piñol V, Balaguer F, Bujanda L, De-Castro ML, Llor X, Andreu M, Carracedo A, Soto JL, Castells A, Jover R. Comparison between universal molecular screening for Lynch syndrome and revised Bethesda guidelines in a large population-based cohort of patients with colorectal cancer. Gut. 2012;doi: 10.1136/gutjnl-2011-300041.
8. Barnetson RA, Tenesa A, Farrington SM, Nicholl ID, Cetnarskyj R, Porteous ME, Campbell H, Dunlop MG. Identification and survival of carriers of mutations in DNA mismatch-repair genesR in colon cancer. N Engl J Med. 2006;doi: 10.1056/NEJMoa053493.
9. Lemos Garcia J, Rosa I, Saraiva S, Marques I, Fonseca R, Lage P, Francisco I, Silva P, Filipe B, Albuquerque C, Claro I. Routine Immunohistochemical Analysis of Mismatch Repair Proteins in Colorectal Cancer-A Prospective Analysis. Cancers (Basel). 2022;doi: 10.3390/cancers14153730.
